# Triptonoterpene, a Natural Product from *Celastrus orbiculatus* Thunb, Has Biological Activity against the Metastasis of Gastric Cancer Cells

**DOI:** 10.3390/molecules27228005

**Published:** 2022-11-18

**Authors:** Haibo Wang, Yuanyuan Luo, Yaqi Hu, Xinyi Feng, Jun Feng, Zewen Chu, Shiya Ou, Xiaojun Dai, Xiaoqing Wang, Yanqing Liu

**Affiliations:** 1 Institute of Translational Medicine, Medical College, Yangzhou University, Yangzhou 225001, China; 2 The Key Laboratory of Syndrome Differentiation and Treatment of Gastric Cancer of the State Administration of Traditional Chinese Medicine, Yangzhou 225001, China; 3 Department of Oncology, Gaoyou Hospital of Traditional Chinese Medicine, Yangzhou 225600, China

**Keywords:** triptonoterpene, natural product, *Celastrus orbiculatus* Thunb, metastasis, gastric cancer

## Abstract

Cancer is one of the greatest threats to human health. Gastric cancer (GC) is the fifth most common malignant tumor in the world. Invasion and metastasis are the major difficulties in the treatment of GC. Herbal medicines and their extracts have a lengthy history of being used to treat tumors in China. The anti-tumoral effects of the natural products derived from herbs have received a great deal of attention. Our previous studies have shown that the traditional Chinese herb *Celastrus orbiculatus* Thunb extract (COE) can inhibit the invasion and metastasis of GC cells, but the specific anti-cancer components of COE are still unclear. Dozens of natural products from COE have been isolated and identified by HPLC spectroscopy in our previous experiments. Triptonoterpene is one of the active ingredients in COE. In this study, we focused on revealing whether Triptonoterpene has an excellent anti-GC effect and can be used as an effective component of *Celastrus orbiculatus* Thunb in the treatment of tumors. We first observed that Triptonoterpene reduces GC cell proliferation through CCK-8 assays and colony formation experiments. The cell adhesion assays have shown that Triptonoterpene inhibits adhesion between cells and the cell matrix during tumor invasion. In addition, the cell migration assay has shown that Triptonoterpene inhibits the invasion and migration of GC cells. The high-connotation cell dynamic tracking experiment has also shown the same results. The effects of Triptonoterpene on epidermal mesenchymal transition (EMT)-related and matrix metalloproteinases (MMPs)-related proteins in gastric cancer cells were detected by Western blots. We found that Triptonoterpene could significantly inhibit the changes in EMT-related and invasion and metastasis-related proteins. Altogether, these results suggest that Triptonoterpene is capable of inhibiting the migration and invasion of GC cells. Triptonoterpene, as a natural product from *Celastrus orbiculatus* Thunb, has significant anti-gastric cancer effects, and is likely to be one of the major equivalent components of *Celastrus orbiculatus* Thunb.

## 1. Introduction

Gastric cancer (GC) is the fifth most common malignancy worldwide and the third leading cause of cancer-related death [1]. The regions with a high incidence of GC mainly include East Asia, Eastern Europe, and South America, and the incidence is generally higher in men than in women [2,3]. People with GC have a low survival rate, and most patients are in the advanced stage when they are diagnosed. The 5-year survival rate of such people is only 20–30% [4,5]. Invasion and metastasis are important malignant characteristics of GC [6,7], and metastatic GC is one of the main causes of death [8]. Inhibiting tumor metastasis can prevent metastatic cancer cells from colonizing other organs of the body, thus delaying the deterioration of tumors [9]. However, current therapeutic measures against GC metastasis are not satisfying, and finding and developing effective drugs or chemicals to inhibit GC metastasis has become one of the focuses of anti-cancer drug development. The most surprising aspect is that as a promising anti-tumoral therapy, Traditional Chinese medicine (TCM), can play an anti-tumoral role by promoting cancer cells’ apoptosis, inhibiting cancer cells’ metastasis, and so forth [10]. Moreover, TCM has the characteristics of multiple targets, minor side effects, and potent efficacy, which can prolong the survival period of patients and improve their quality of life [11]. Many studies have shown that TCM has good therapeutic effects on GC [12,13,14]. *Celastrus orbiculatus* Thunb extract (COE) is an anti-tumoral ingredient from Chinese medicine that has been studied by our research group for a long time. *Celastrus orbiculatus* Thunb is a species in the genus Celastrus (family: Celastraceae). The extraction and use of the effective components of COE have been authorized by the national invention patent (Grant No: 200710025343.3). The previous research results showed that COE could inhibit EMT, invasion, and the metastasis of GC cells [15,16,17,18]. However, the main anti-GC components of COE are still being explored. In our early-stage studies, we have separated and identified 26 active components from COE and speculated that Triptonoterpene may have an anti-GC effect according to the preliminary screening results [19,20]. This study focused on investigating whether Triptonoterpene, one of the active ingredients of COE, produces anti-tumoral effects, thereby revealing the main anti-tumoral component of COE. This will help to lay a more solid theoretical and experimental foundation for COE as an anti-tumoral TCM agent.

## 2. Results

### 2.1. Triptonoterpene Inhibits the Viability of Gastric Cancer Cells

**Figure 1:**
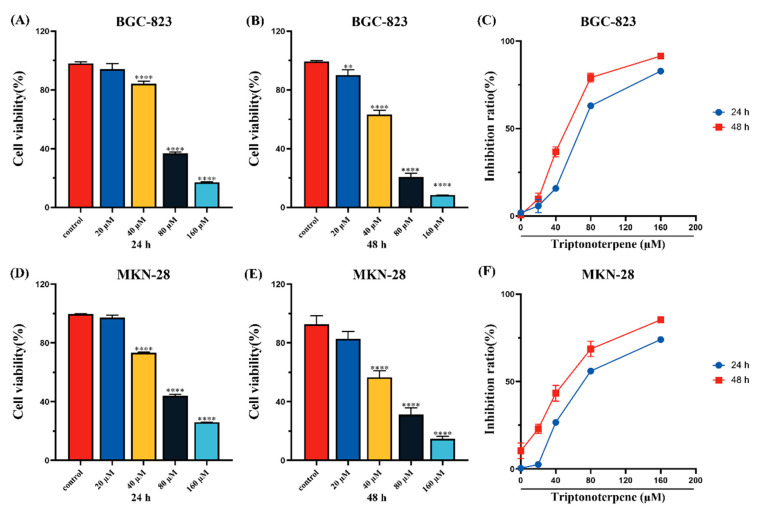
Cell viability was detected using the CCK-8 assay. (**A**,**B**) The survival rates of the BGC-823 cells after 24 h and 48 h interventions with different concentrations of Triptonoterpene. (**C**) The inhibitory rates of Triptonoterpene on the BGC-823 cells after 24 h and 48 h. (**D**,**E**) The survival rates of the MKN-28 cells after 24 h and 48 h interventions with different concentrations of Triptonoterpene. (**F**) The inhibitory rates of Triptonoterpene on the MKN-28 cells after 24 h and 48 h. ** *p* < 0.01 and **** *p* < 0.0001.

The CCK-8 experiment’s results show that compared with the control group, the viability of the GC cells treated with Triptonoterpene (0, 20, 40, 80, and 160 μM) was inhibited in concentration- and time-dependent manners (Figure 1). IC_50_ values were calculated with GraphPad Prism 8.0 software. The IC_50_ values of the BGC-823 cells treated with Triptonoterpene for 24 h and 48 h were 62.84 μM and 47.74 μM, respectively. The IC50 values of the MKN-28 cells treated with Triptonoterpene for 24 h and 48 h were 56.16 μM and 42.56 μM, respectively. In order to avoid the toxic effects of Triptonoterpene on the BGC-823 and MKN-28 cells and to better demonstrate Triptonoterpene’s anti-tumoral efficacy, the low and medium concentrations (0, 20, 40, and 80 μM) and a 24 h treatment time were selected in the subsequent experiments to explore the inhibitory effects of Triptonoterpene on the BGC-823 cells and MKN-28 cells.

### 2.2. Triptonoterpene Inhibits the Proliferation of Gastric Cancer Cells

**Figure 2:**
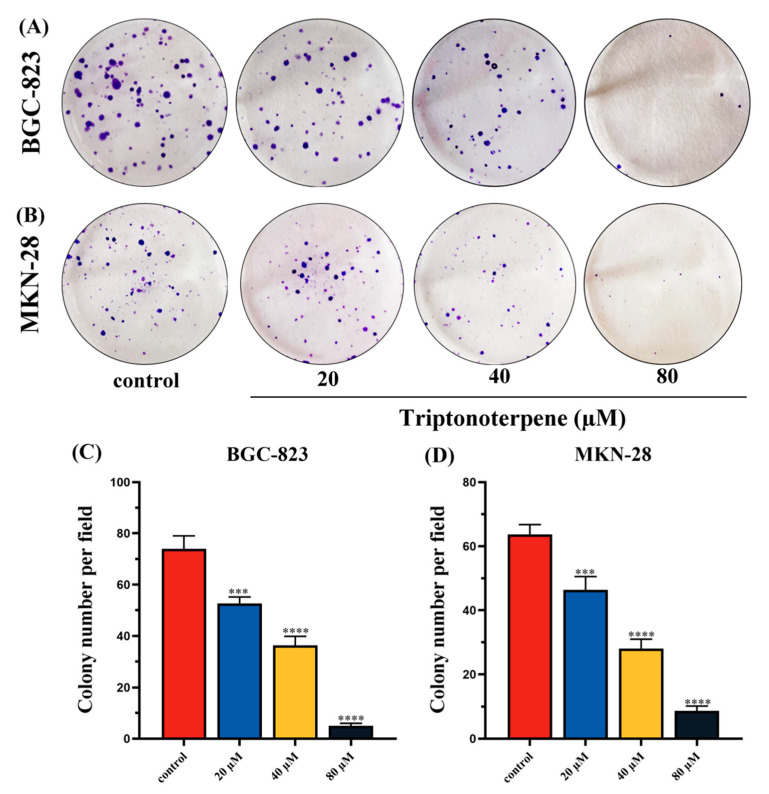
Cell proliferation was examined by the cell colony formation experiment. (**A**,**C**) Number of clones of the BGC-823 cells treated with Triptonoterpene for 24 h. (**B**,**D**) Number of clones of the MKN-28 cells treated with Triptonoterpene for 24 h. *** *p* < 0.001 and **** *p* < 0.0001.

The effects of Triptonoterpene on the proliferation of the BGC-823 cells and MKN-28 cells were observed through colony formation experiments. The experimental results show that the number of colonies formed for the Triptonoterpene group cells was significantly reduced (Figure 2), suggesting that Triptonoterpene can effectively inhibit the proliferation of the GC cells. These results also preliminarily prove that Triptonoterpene has anti-tumoral activity, and it may be one of the active anti-tumoral ingredients of COE.

### 2.3. Triptonoterpene Inhibited the Adhesion of Gastric Cancer Cells to Cell Matrix

**Figure 3:**
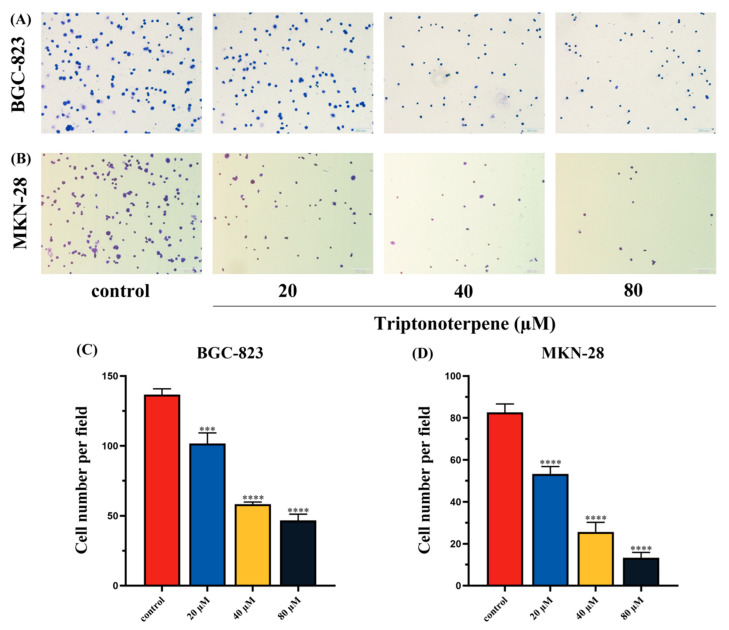
The cell adhesion ability detected by the cell adhesion experiment. (**A**) The number of cell adhesions observed under a microscope after the 24 h treatment of Triptonoterpene applied to the BGC-823 cells. (**B**) The number of cell adhesions observed under a microscope after the 24 h treatment of Triptonoterpene applied to the MKN-28 cells. (**C**) Statistical chart of the adhesion number of the BGC-823 cells. (**D**) Statistical chart of the adhesion number of the MKN-28 cells. *** *p* < 0.001 and **** *p* < 0.0001.

In order to explore the effect of Triptonoterpene on the invasion and metastasis of the GC cells, cell adhesion experiments were performed to assess the adhesion ability of Triptonoterpene to the BGC-823 and MKN-28 cell stroma. The experimental results show that the number of adhesions of the Triptonoterpene group cells was significantly reduced (Figure 3). This finding suggests that Triptonoterpene can inhibit cells’ adhesion to the cell matrix at the beginning of cell invasion.

### 2.4. Triptonoterpene Inhibits the Migration of Gastric Cancer Cells

**Figure 4:**
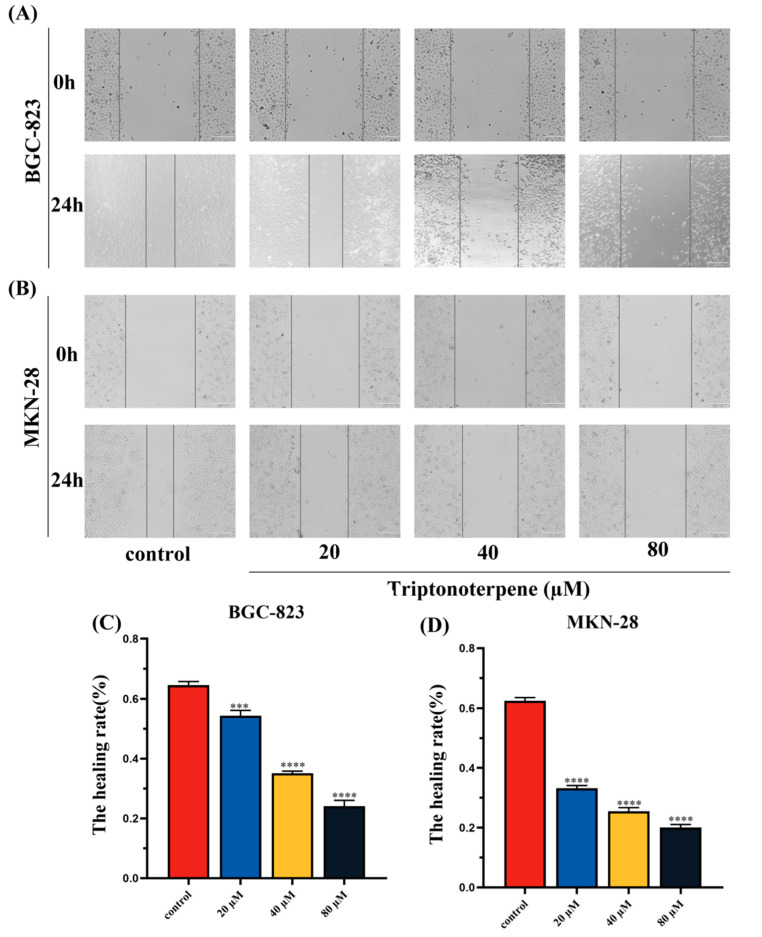
The cell migration capacity was measured using the wound healing assay. (**A**) Microscopic wound area of the BGC-823 cells. (**B**) Microscopic wound area of the MKN-28 cells. (**C**) Wound-healing rate of the BGC-823 cells. (**D**) Wound-healing rate of the MKN-28 cells. *** *p* < 0.001 and **** *p* < 0.0001.

Wound-healing experiments were performed to detect the effects of Triptonoterpene on the GC cells’ migration. The results show that the cells of the Triptonoterpene group had a larger wound area and a lower wound-healing rate (Figure 4). This finding suggests that Triptonoterpene can inhibit the migration of GC cells.

### 2.5. Triptonoterpene Inhibits the Invasion of Gastric Cancer Cells

**Figure 5:**
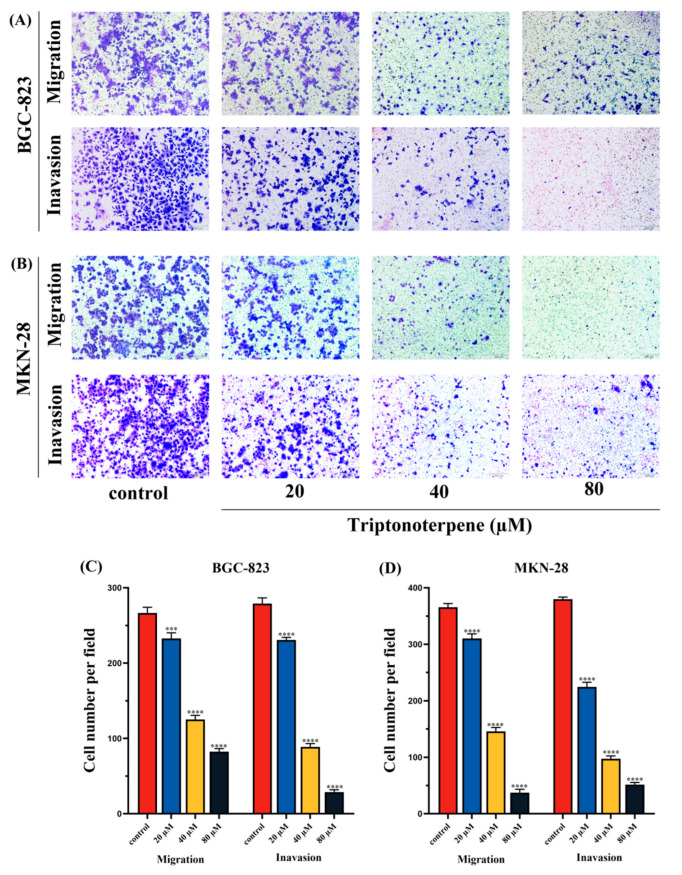
The invading and metastatic ability of the GC cells was examined using the Transwell assay. (**A**) Number of the BGC-823 cells migrating and invading under the microscope. (**B**) Number of the MKN-28 cells migrating and invading under the microscope. (**C**) Statistical chart of the number of migrating and invading BGC-823 cells. (**D**) Statistical chart of the number of migrating and invading MKN-28 cells. *** *p* < 0.001 and **** *p* < 0.0001.

To further determine the effects of Triptonoterpene on the invasion and metastasis of the BGC-823 cells and MKN-28 cells, Transwell experiments were carried out. The experimental results show that Triptonoterpene can significantly reduce the number of invading and migrating GC cells (Figure 5). The above findings demonstrate for the first time that Triptonoterpene can inhibit the invasion and metastasis of GC cells.

### 2.6. Triptonoterpene Inhibits the Dynamic Migration of the Gastric Cancer Cells

**Figure 6:**
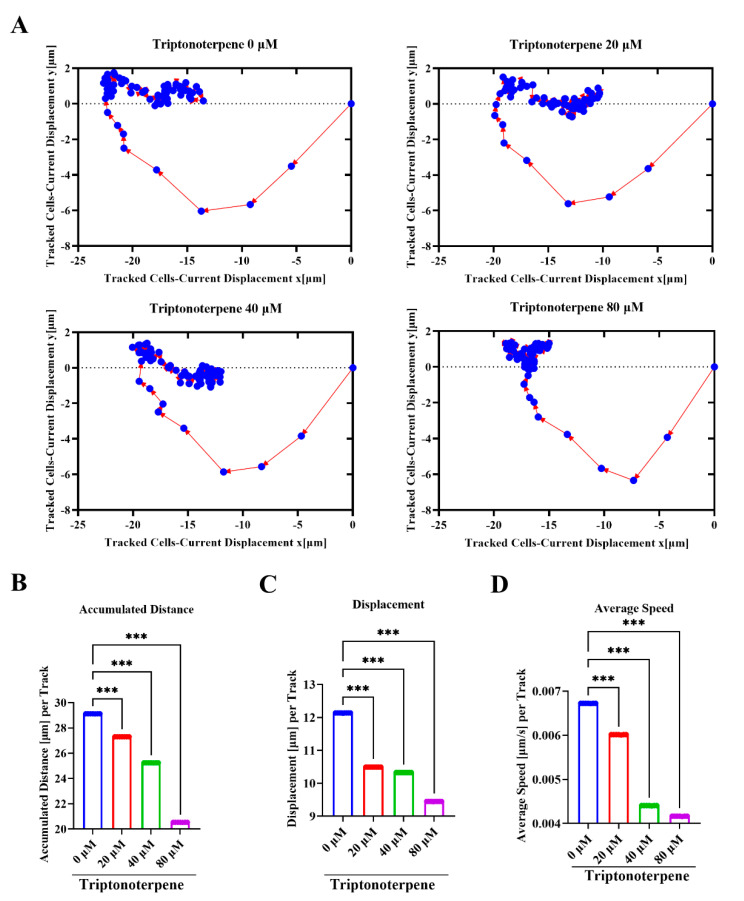
The movement tracks and speed of the GC cells were examined using high−intensity imaging technology. (**A**) Locus of GC cells 12 h after the Triptonoterpene treatment. (**B**) Total movement distance of the GC cells in 12 h. (**C**) Average displacement of the GC cells in different drug groups. (**D**) Average movement speed of the GC cells in different drug groups within 12 h. *** *p* < 0.001.

In order to verify the effect of Triptonoterpene on the motility of GC cells, we dynamically tracked the motility of the GC cells within 12 h using high-intensity technology. We mapped the movement tracks of the GC cells within 12 h and found that the range of the GC cell-related movement paths of the Triptonoterpene group decreased. (Figure 6A). At the same time, the total migration distance and displacement of the GC cells of the Triptonoterpene group were also shortened (Figure 6B,C). In addition, the high-intensity imaging technology also tracked the changes of the GC cells’ movement speed. We found that the average movement speed of the GC cells in the Triptonoterpene group was lowered (Figure 6D). These results confirm that Triptonoterpene can inhibit the migration of GC cells.

### 2.7. Triptonoterpene Affects the Expression of the EMT-Related Proteins in Gastric Cancer Cells

**Figure 7:**
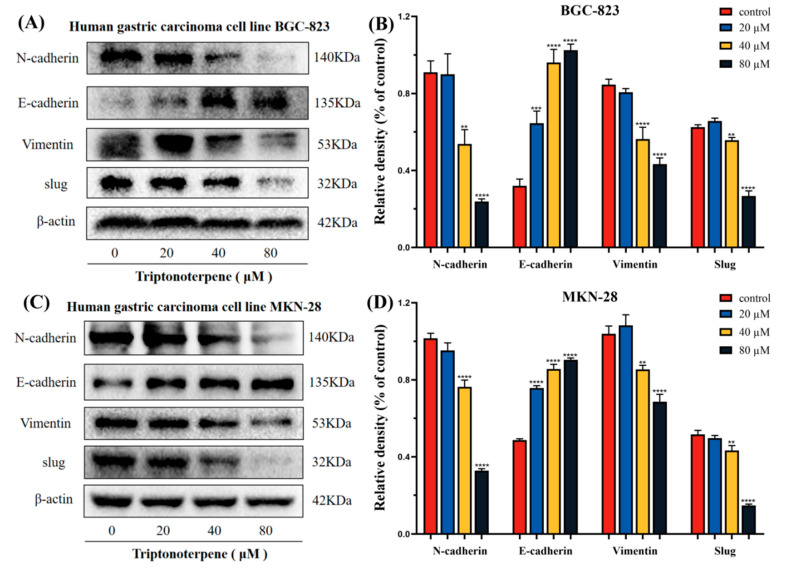
The expression levels of the EMT-related proteins in the GC cells was detected using Western blots. (**A**,**B**) Expression levels of N-cadherin, E-cadherin, Vimentin, and Slug in the BGC-823 cells. (**C**,**D**) Expression levels of N-cadherin, E-cadherin, Vimentin, and Slug in the MKN-28 cells. ** *p* < 0.01 and **** *p* < 0.0001.

To further explore the mechanism whereby Triptonoterpene inhibits the GC cells’ invasion and metastasis, we measured the expression levels of the EMT-related proteins. The expression levels of these proteins directly reflect the invasion ability of the GC cells. The experimental results show that Triptonoterpene upregulates the expression level of E-cadherin in the GC cells, while the expression levels of N-cadherin, Vimentin, and Slug are down-regulated (Figure 7). The Western blot results suggest that Triptonoterpene may be able to inhibit GC cell invasion and metastasis by inhibiting the EMT process.

### 2.8. Triptonoterpene Affects the Expression of the MMP-Related Proteins in the Gastric Cancer Cells

**Figure 8:**
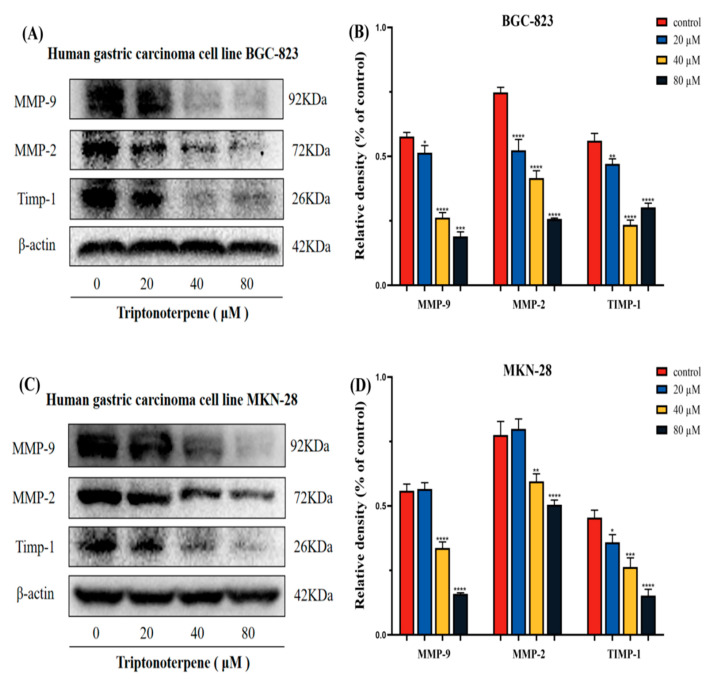
The expression of the MMP-related proteins in the GC cells was detected using Western blots. (**A**,**B**) Expression levels of MMP-9, MMP-2, and Timp-1 in the BGC-823 cells. (**C**,**D**) Expression levels of MMP-9, MMP-2, and Timp-1 in the MKN-28 cells. * *p* < 0.05, ** *p* < 0.01, *** *p* < 0.001, and **** *p* < 0.0001.

We also examined the effects of Triptonoterpene on the expression of the key proteins of MMPs. The experimental results show that the expression of MMP-9, MMP-2, and Timp-1 of the Triptonoterpene group were reduced in the BGC-823 and MKN-28 cells (Figure 8). This shows that Triptonoterpene can inhibit the expression of key proteins of MMPs to prevent the degradation of ECM.

## 3. Discussion

*Celastrus orbiculatus* Thunb is an anti-tumoral ingredient in TCM that has been studied by our project group for a long time. In our early research results, the anti-tumoral efficacy of COE has been confirmed [21,22,23,24,25,26], but the specific anti-tumoral component of COE is unclear. We isolated and identified 26 effective monomers from COE and preliminarily found that Triptonoterpene may inhibit GC cell growth. This study aims to explore the effects of Triptonoterpene on the invasion and metastasis of GC cells in order to determine the effective anti-tumoral composition of COE. The preliminary results from the CCK-8 trials found that Triptonoterpene may have an anti-tumoral effect. The colony formation experiments further confirmed that Triptonoterpene could inhibit the proliferation of GC cells. This suggests that Triptonoterpene has anti-cancer potential, so we further explored the effects of Triptonoterpene on GC metastasis. The tumor metastasis process includes several main phases such as adhesion, motility, and invasion. In particular, the loss of E-cadherin expression is thought to be an important step in carcinogenesis. In our study, we found that Triptonoterpene can up-regulate the expression of E-cadherin. We realized that Triptonoterpene may have similar effects to COE in inhibiting the invasion and metastasis of GC cells. To further explore the effect of Triptonoterpene on the metastasis of the GC cells, we observed the effect of Triptonoterpene on cell adhesion through cell adhesion experiments. The experimental results show that Triptonoterpene significantly inhibits cell and cell matrix adhesion. The adhesion of tumor cells is a key step in tumor invasion and metastasis [27]. Before metastasis occurs, tumor cells need to adhere to the cell matrix after separating from the mother cells. Thus, the inhibition of tumor cell adhesion suppresses the occurrence of tumor cell invasion and metastasis. This result suggests that Triptonoterpene inhibits cell–cell matrix adhesion at the onset of cell metastasis. After contacting the extracellular matrix, tumor cells must secrete enzymes to degrade and cross the ECM barrier. Currently, MMPs are recognized as the most important enzymes for ECM degradation, and in particular, the abnormal expression of MMP-2 and MMP-9 can lead to accelerated tumor progression. We detected that Triptonoterpene was able to reduce the expression levels of the MMP-9/2 protein. This result indicates that Triptonoterpene can prevent the first line of defense against tumor invasion and metastasis from being destroyed. It is important to note that MMPs also affect cell adhesion, and the inhibition of MMPs expression also suppresses the adhesion of tumor cells to the extracellular matrix.

Cell movement is essential for tumor cells’ metastasis. Cancer cells must first have the ability to move in order to metastasize, meaning that cancer cell movement is a prerequisite for cancer cells ability to attack surrounding tissue. Through the wound-healing assay, Transwell assay, and other functional experiments, we found that Triptonoterpene could inhibit the invasion and migration of the GC cells. In the present study, Triptonoterpene’s ability to inhibit the dynamic migration of the GC cells was clearly observed by high-intensity imaging. Our results also fully demonstrate that key steps in tumor metastasis can be inhibited by Triptonoterpene. Inhibiting tumor invasion and migration is a feasible method for anti-tumoral treatment [28]. To additionally verify the effects of Triptonoterpene on the invasion and metastasis of GC cells, we detected the changes in EMT- and metastasis-related proteins. EMT is related to occurrence, invasion, and metastasis [29,30,31]. Our previous study found that COE could inhibit the invasion and metastasis of GC cells by increasing E-cadherin levels and decreasing N-cadherin and Vimentin expression [18]. In this study, we found that Triptonoterpene had effects similar to COE on these proteins in the GC cells, not only up-regulating the expression of E-cadherin but also down-regulating the expression of N-cadherin and Vimentin. In addition, Triptonoterpene can also reduce the expression of Slug in the GC cells. Slug can participate in the inhibition of E-cadherin expression in GC, and Slug expression is negatively correlated with the E-cadherin expression [32]. These results also confirmed that Triptonoterpene may be one of the effective anti-tumoral components of COE. Taken together, Triptonoterpene, as a natural product from *Celastrus orbiculatus* Thunb, has significant anti-gastric cancer effects, and is likely to be one of the essential equivalent components of *Celastrus orbiculatus* Thunb. This study lays a solid foundation for uncovering the specific molecular composition and anti-tumoral mechanisms of *Celastrus orbiculatus* Thunb.

## 4. Materials and Methods

### 4.1. Drug

COE is ethyl acetate-extracted from a part of *Celastrus orbiculatus* Thunb and is a crude plant extract. The detailed process of drug extraction was described previously [19,33]. Triptonoterpene is a natural product isolated from COE. In order to meet the requirement of experimental purity, we purchased commercial Triptonoterpene for experiments. Triptonoterpene (standard substance; HPLC ≥ 98%) (Shanghai Yuanye Biotechnology, Shanghai, China) was dissolved in dimethyl sulfoxide (DMSO) at a concentration of 100 mM, and then diluted to the required drug concentrations for use.

### 4.2. Reagents

The following reagents were employed in this study: RPMI Medium Modified (HyClone, Waltham, MA, USA); trypsin (Solarbio, Beijing, China); fetal bovine serum (FBS; TransSerum^®^ FQ Fetal Bovine Serum, Beijing, China); DMSO (Sigma, Berlin, Germany); Transwell-permeable supports and 8.0 μm polycarbonate membranes (Corning, New York, NY, USA); Cell Counting Kit-8 and Crystal Violet Staining Solution (Beyotime Biotechnology, Shanghai, China); MMP-2, MMP-9, E-cadherin, N-cadherin, Vimentin and β-actin (Thermo Fisher Scientific, Waltham, MA, USA); TIMP-1 (Abcam, Cambridge, UK); anti-rabbit IgG, HRP-linked antibody, Slug (CST, Danvers, MA, USA).

### 4.3. Cell Culture

BGC-823 and MKN-28 cells were cultured in 1640 medium containing 10% FBS. The cells were cultured in a 37 °C incubator containing 5% CO_2_, and the fresh culture medium was changed once every 2–3 days. When the degree of cell fusion reached more than 80% it was digested with trypsin for passage.

### 4.4. Cell Viability Assay

The BGC-823 and MKN-28 cells were seeded in 96-well plates at 3000 cells per well. After the cells adhered to the wall, the GC cells were intervened with different concentrations of Triptonoterpene (0, 20, 40, 80, and 160 μM). Then, 10 μL CCK-8 solution was added to each well after the cells were treated with Triptonoterpene for 24 h and 48 h. The 96 well plates were placed in the incubator and incubated for 2 h. An EnSpire microplate reader was used to read the absorbance (A) value of cells at 450 nm. Half inhibitory concentration (IC_50_) was calculated for the subsequent experiments.

### 4.5. Colony Formation Assay

The BGC-823 cells and MKN-28 cells were inoculated into 6-well plates at 1000 cells per well. When the cells adhered to the wall and began to proliferate, different concentrations of Triptonoterpene were added to the wells. The cells were cultured in the drug-containing medium for 2 weeks, and the culture was terminated when the cells showed a visible colony. The cells were washed with PBS and fixed with methanol for 30 min. Then, the cells were stained with crystal violet for 15 min, and the number of colonies was observed and photographed.

### 4.6. Cell Adhesion Assay

The BGC-823 cells and MKN-28 cells were treated with Triptonoterpene for 24 h. the Matrigel was diluted 8 times by the serum-free medium, and the diluted Matrigel was spread onto 24-well plates. After the Matrigel in the 24-well plates solidified, PBS was used to clean the excess glue. The Triptonoterpene-treated GC cells were then seeded in the 24-well plates with 2 × 10^4^ cells per well and cultured for 60 min. The medium was discarded, and the cells were fixed with methanol for 30 min and stained with crystal violet for 15 min. Images from five fields of view were randomly captured under an inverted microscope.

### 4.7. Wound Healing Assay

The BGC-823 and MKN-28 cells were seeded at 3 × 10^5^ cells per well in a 24-well plate. When the cells’ fusion degree exceeded 80%, linear scratch wounds were created on the confluent cell monolayers using a 200 μL pipette tip. Triptonoterpene was added to the cells and the cells were incubated at 37 °C in a 5% CO_2_ incubator. After Triptonoterpene was added to the GC cells, the scratch area of the cells was observed and photographed under a microscope at 0 h and 24 h, respectively.

### 4.8. Transwell Chamber Assay

First, 50 μL Matrigel gel was added to the Transwell chambers, which solidified at 4 °C. The BGC-823 and MKN-28 cells were resuspended in serum-free medium and added to the upper chamber at 2 × 10^4^ cells per well. Triptonoterpene was added into the lower chamber and the cells were continuously cultured for 24 h. The medium was discarded, 4% paraformaldehyde was used to immobilize cells for 15 min, and then crystal violet was used to dye cells for 15 min. After the chamber was cleaned with PBS, the number of cells penetrating the membrane was observed under an inverted microscope. The cell migration assay did not require the addition of Matrigel to the 24-well plate. The other steps of the cell migration assay are the same as those of the cell invasion assay.

### 4.9. Operetta CLS High-Content Cell Dynamic Tracking Assay

The MKN-28 cells were seeded in 96-well plates at 3000 cells per well. The GC cells were treated with different concentrations of Triptonoterpene for 12 h after cell adhesion. The 96-well plates were placed in a high-content analysis system for dynamic tracking for 12 h. The movement trajectory, moving distance, and moving speed of the cells were recorded within this time period.

### 4.10. Western Blot Analysis

The BGC-823 and MKN-28 cells were treated with different concentrations of Triptonoterpene for 24 h to extract total protein. The proteins were separated by SDS-PAGE electrophoresis and transferred to polyvinylidene fluoride (PVDF) membranes. Sealing liquid was used to seal the PVDF membranes for 2 h. Primary antibodies were diluted and incubated with the membranes for 12 h at 4 °C. Then, fluorescence secondary antibodies were added and incubated with the membranes for 2 h. The bands were detected and quantified using a Bio-Rad imaging system.

### 4.11. Statistical Analysis

All data were averaged from at least three independent trials. The data within the group conformed to a normal distribution. Ordinary one-way ANOVA and multiple comparative analysis were performed with Graph Prism 8.0 software. *p* < 0.05 was considered to denote statistical significance.

## 5. Conclusions

The innovation uncovered in this study is that Triptonoterpene can inhibit the invasion and metastasis of GC cells. These results confirmed that Triptonoterpene may be one of the effective anti-tumoral components of COE. Furthermore, Triptonoterpene, as a natural product from *Celastrus orbiculatus* Thunb, has significant anti-GC effects, and is probably one of the important equivalent components of *Celastrus orbiculatus* Thunb. This research will lay a solid foundation for uncovering the specific molecular composition and anti-tumoral mechanisms of *Celastrus orbiculatus* Thunb.

## Data Availability

All data included in this study are available upon request by contact with the corresponding author.

## Sample Availability

Samples of the compounds are available from the authors.
